# Is physical restraint unethical and illegal?: a qualitative analysis of Korean written judgments

**DOI:** 10.1186/s12912-024-01781-8

**Published:** 2024-02-04

**Authors:** Seung Gyeong Jang, Won Lee, Jeongmin Ha, Sungkyoung Choi

**Affiliations:** 1 Department of Nursing, Inje University, Busan, Republic of Korea; 2 Department of Nursing, Chung-Ang University, Seoul, Republic of Korea; 3 Department of Nursing, Dong-A University, Busan, Republic of Korea; 4 Department of Nursing, Catholic Kwandong University, 24, Beomil-ro 579, 25601 Gangneung, Gangwon-do Republic of Korea

**Keywords:** Restraint, physical, Ethical dilemmas, Empirical literature, Dissent and disputes, Jurisprudence, Malpractice

## Abstract

**Background:**

Physical restraint (PR) is used to ensure the safety of care recipients. However, this causes an ethical dilemma between the autonomy and dignity of the recipients and the provision of effective treatment by health workers. This study aimed to analyze legal and ethical situations related to the use of PR using written judgments.

**Methods:**

This study uses a qualitative retrospective design. Qualitative content analysis was performed on South Korean written judgments. A total of 38 cases from 2015 to 2021 were categorized. The types of court decisions and ethical dilemma situations were examined according to the four principles of bioethics, and the courts’ judgments were compared.

**Results:**

Written judgments related to PR were classified into three types according to the appropriateness of PR use, the presence or absence of duty of care, and legal negligence. Ethical dilemmas were categorized into three situations depending on whether the four principles of bioethics were followed. The courts’ decisions regarding the ethical dilemmas differed depending on the situational factors before and after the use of PR and the conflicting conditions of the ethical principles.

**Conclusions:**

Health workers should consider legal and ethical requirements when determining whether to use PR to provide the care recipient with the necessary treatment.

**Supplementary Information:**

The online version contains supplementary material available at 10.1186/s12912-024-01781-8.

## Background

Physical restraint (PR) is a measure that restricts one’s freedom of movement [1] using a wrist strap, abdominal belt, or ankle brace [2]. It is primarily used for safety reasons in individuals [3, 4] with low cognitive function or high care dependency [5, 6], such as older adults with cognitive impairment or a high risk of falls [7], children with decision-making difficulties [8], patients with mental illnesses [9, 10], and critically ill [11] or emergency patients [12] attached to life-sustaining or monitoring devices. However, even if PR is used with good intentions, it has a risk of causing physical and psychological damage [13] to care recipients, and it can sometimes be abused [14] for the convenience of health workers. In particular, nurses are the traditional PR decision-makers who are most involved in patient care [15, 16], but fear of safety accidents, consequent criticism, and legal responsibility contribute to nurses’ use of PR [16], as does individual nurses’ poor ethical sensitivity [17]. All people have the right to equal freedom and dignity [18]; however, PR has long been controversial because it infringes on individuals’ freedom of movement, forcibly restricts autonomy, and has negative impacts on health workers, who experience guilt and ethical dilemmas [10, 12, 19, 20].

In ethical decision-making, the four principles of biomedical ethics (beneficence, non-maleficence, autonomy, and justice) serve as a framework [21]. These principles have been pivotal in establishing the legitimacy of mandatory childhood vaccinations [22] and resolving ethical quandaries in cutting-edge fields such as facial recognition [23]. However, PR presents challenges in situations where patient safety [24] and treatment purposes come into conflict with individuals’ autonomy, or where ethical and legal considerations clash. To provide clarity, guidelines have been established for the use of PR [25], which advocate its deployment as a last resort and employ minimal deterrence to care recipients [26].

Although countries have implemented standards for PR use, variations exist in the type, frequency, and duration of restraint [27]. Consequently, differing perceptions and attitudes towards PR have emerged, making its systematic use increasingly difficult to justify. PR is considered unreasonable when it restricts freedom of movement, involves informal methods such as pressure or threats [14], or is used in response to understaffing and heavy workloads [28]. For reference, in South Korea, there was an incident in 2014 where a fire broke out in a long-term care hospital, resulting in the tragic death of numerous elderly patients who were tied to their beds without being rescued. Afterwards, the standards for PR use were included in the Medical Service Act, making compliance with the standards mandatory. This act stipulates the following: criteria for PR use, minimization of PR use, restraint device that can be easily released or cut in an emergency, and duties of physicians and nurses (periodic observation of patients using PR, provision of interventions to meet patients’ needs, and recording).

A written judgment contains factual information about a specific dispute, the arguments of both parties (the plaintiff and defendant), and the court’s decision regarding the dispute [9, 29]. These judgments facilitate empirical research on cases obscured by confidentiality or personal data concerns [30]. Courts’ decisions provide valuable information that can only be found within written judgments; they play a crucial role in upholding societal order and establish legal precedents that are binding in countries adhering to the common law system [31].

External observation of PR is restricted owing to its predominant usage in enclosed environments such as intensive care units, psychiatric wards, and long-term care facilities. Therefore, the information contained in written judgment can be used to address PR dilemmas. Clear legal and ethical standards are essential for implementing PR. Many previous studies have focused on PR guidelines [25, 32] and effectiveness [4, 26]; however, there are few studies that analyze the legitimacy and results of PR judgments. Thus, this study aimed to provide empirical data for health workers that can be used to determine reasonable and appropriate PR use in clinical settings. In this regard, this study analyzes PR-related situations through written judgments using the four principles of bioethics and examines courts’ decisions for ethical dilemmas.

## Methods

### Study design

This qualitative retrospective study utilized content analysis on written judgments containing information about litigation cases related to PR in South Korea, and was guided by the four principles of biomedical ethics proposed by Beauchamp and Childress [21].

### Data collection

**Fig. 1:**
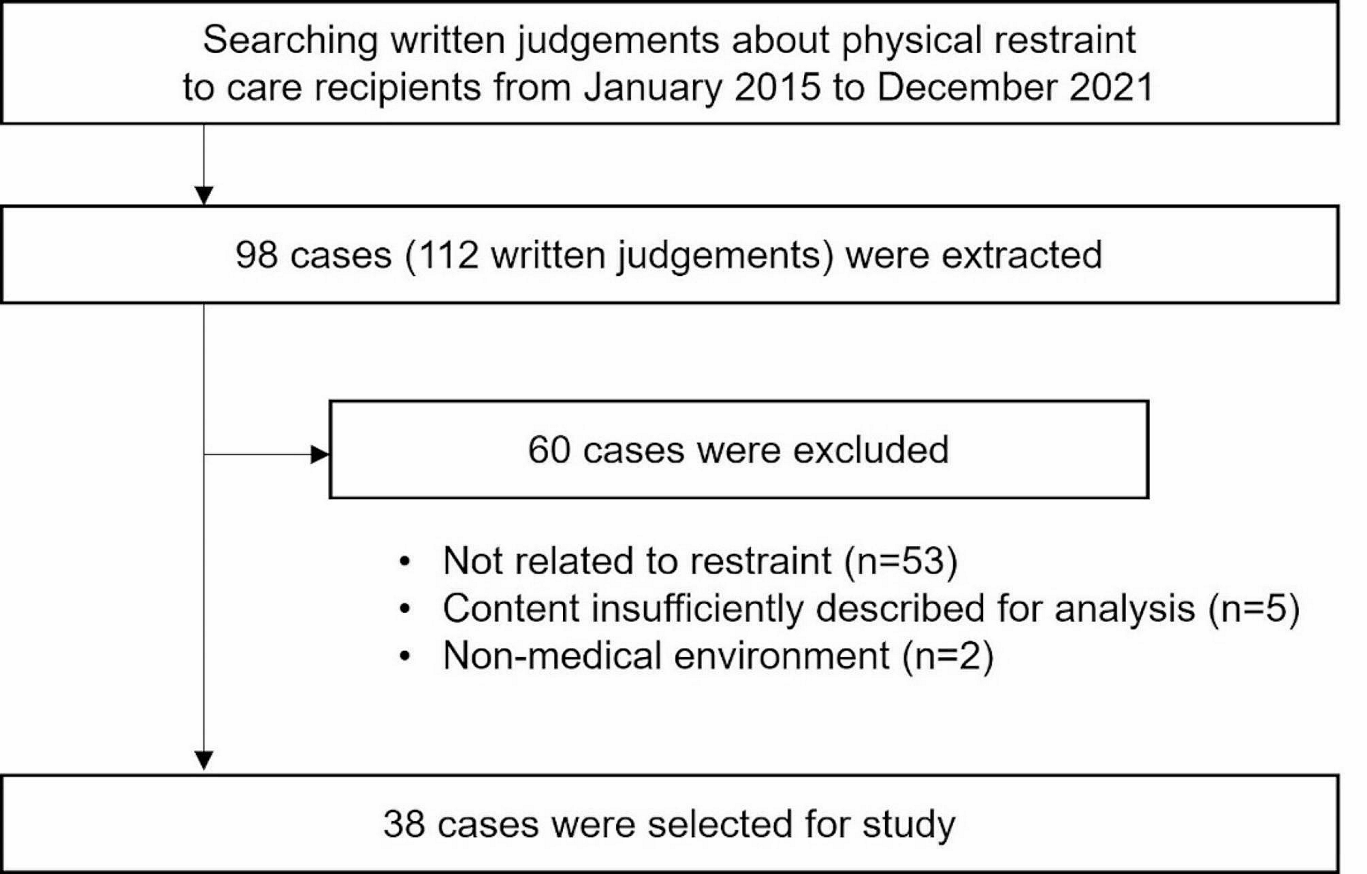
Data selection flow chart

Written judgments were collected from the “Written Judgment Management System” run by the Supreme Court of South Korea. Anyone in South Korea can access this system online and can search for and read judgments with redacted personal information. In December 2021, we searched for judgments in this system using the keyword “restraint.” The search period was from January 2015 to December 2021, when written judgments were released to the public. One hundred and twelve written judgments were extracted, and we classified the written judgments based on the “case” to which they belonged, because a maximum of three written judgments can exist for one case (the three-trial system). In these judgments, a total of 98 cases were identified. SGJ read the retrieved written judgments and reviewed whether the cases fit the purpose of the study, while SC confirmed the results of the review. Cases that were not appropriate for the purposes of this study were excluded. Finally, 38 cases were included in the study (Fig. 1).

### Data analysis

Qualitative content analysis is a research method that analyzes text data to describe a specific phenomenon that a researcher wants to explore [33, 34]. Data analysis was conducted according to the qualitative content analysis process of Elo and Kyngas [33].

#### Development of coding matrix and analysis framework

We clarified the purpose of the study and discussed which information to identify in written judgments [30, 35] for the first step. Three categories were organized as elements for content analysis: basic information about the lawsuit and the case, and court’s decision. Detailed description of each category is presented in supplementary material. We developed a coding matrix that listed to the 38 cases and categories to identify information without omissions according to these categories. Additionally, we developed an analytical framework using the four principles of Beauchamp and Childress [21] to classify and analyze lawsuit cases from an ethical perspective (Table 1) These four principles were designed to help resolve ethical issues using the concepts of beneficence, non-maleficence, autonomy, and justice [21]. To develop the framework, we searched for previous studies on PR and held regular meeting to reach a consensus. As a result, the criteria for determining compliance with ethical principles in PR-related cases were established, and three ethical dilemmas that can arise when using PR were identified.

**Table 1:** Criteria for ethical dilemma situation assessment grounded in the four ethical principles

Ethical principles	Criteria			Ethical dilemma situations		
	Definition /Meaning	Compliance	Violation	1 Can PR be used without consent for safety?	2 Is it possible to use PR due to staff shortage? (Without consent)	3 Is it possible to use PR due to staff shortage? (With consent)
**Beneficence**	Was the decision to use or not to use PR the best choice for the recipient?	The court determined that the judgment of the health worker regarding the use of PR was not illegal or negligent.	The court decided the judgment of the health worker regarding the use of PR as illegal or negligent.	C	-	-
**Autonomy**	Were the recipients or their guardians informed about the use of PR and did they write the consent?	PR was applied after obtaining prior consent.	PR was used without prior consent.	V	V	C
**Justice**	Was the allocation of medical resources fair in relation to PR?	No relevant cases available.	PR was not used due to a lack of resources like staff and materials.	-	V	V
**Non-maleficence**	Did any harm occur to the recipients due to the use or non-use of PR?	The judgment of the health worker regarding the use of PR did not result in any adverse outcomes for the recipient.	The judgment of the health worker regarding the use of PR resulted in adverse outcomes for the recipients.	-	-	-

Note: PR = Physical restraint; C = compliance; V = violation; Violations of the ‘principle of justice’ were recognized only if the written judgments specified the content of lack of medical resources (e.g., staff shortage)

#### Coding and analysis

SGJ and SC independently read the written judgments and entered the information identified within into the coding matrix according to the data analysis criteria. They reread a few times as necessary to fully understand the case. To check for disagreements and prevent the omission of information to ensure accuracy, the matrices coded by each researcher were exchanged and inspected. Subsequently, SC and SGJ assembled and utilized the data within the matrix to evaluate each of the 38 cases’ compliance with the four ethical principles, as per the analytical framework. Cases corresponding to the ethical dilemmas in the analytical framework were selected. Subsequently, three research meetings were convened, during which the analysis outcomes of SC and SGJ were collectively reviewed and agreed upon. Furthermore, during the analysis, behaviors related to the use of PR were closely linked to a series of nursing processes (assessment, diagnosis, planning, implementation, and evaluation). The results of this study were synthesized into a flowchart based on these nursing processes.

### Ethical considerations

This study was reviewed and deemed exempt by the Institutional Review Board (IRB) of Chung-Ang University as written judgements are available to the public and are de-identified (IRB no. 1041078-202111-HR-333-01; exemption granted under Article 15 of South Korea’s Bioethics and Safety Act). In accordance with the IRB exemption approval, the need for informed consent to participate was also waived.

## Results

### General characteristics

The general characteristics of the 38 cases are shown in Table 2. Most cases related to PR occurred in hospitals (*n* = 24), followed by nursing homes (*n* = 12). Twenty-two care recipients had cognitive deficits due to dementia, mental illness, or brain damage, while four required medically essential devices, such as endotracheal tubes and intravenous lines. In total, 10 care recipients had both. Healthcare assistants, including long-term care nursing assistants and caregivers of older adults, constituted the largest group (*n* = 19) of healthcare workers who provide care on the frontline, followed by health professionals such as physicians and nurses (*n* = 17). Cases were categorized as the use of PR (*n* = 27) or disuse of PR (*n* = 11). In the PR cases, four care recipients fell off the bed or wheelchair as the restraints were loosened, three self-removed medically necessary devices as the restraints were loosened, and five died from fire, pulmonary arterial thrombosis, cardiorespiratory arrest, or suicide. In the non-PR cases, seven care recipients fell from beds or wheelchairs and three self-removed medically necessary devices.

**Table 2:** General characteristics of the cases (*n* = 38)

Characteristics	Number
**Place**	
Hospital	24
General ward	9
Intensive care unit	5
Psychiatric ward	5
Long-term care hospital	4
Emergency room	1
Nursing home	12
Ambulance	1
Residential homes for people with intellectual disability	1
**Care recipient’s condition**	
Cognitive deficits	22
Medical tubes, lines, and catheters	4
Both	10
Not applicable	2
**Frontline health workers**	
Health professionals	17
Health care assistants	19
Ambulance workers	1
Care staff in the residential homes for people with intellectual disability	1
**Consequences of care recipients**	
Cases on the use of PR (*n* = 27)	
Fall	4
Self-removal of medical tubes, lines, and catheters	3
Expire	5
Nothing happened	3
No information	12
Cases on the disuse of PR (*n* = 11)	
Fall	7
Self-removal of medical tubes, lines, and catheters	3
Expire	0
Nothing happened	1

Note: PR = physical restraint

### Courts’ decisions for cause of action

**Fig. 2:**
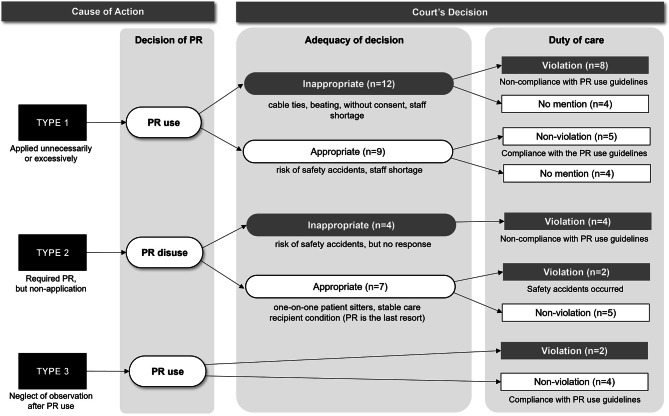
Types of court’s decision for the cause of action. *Note*: PR = physical restraint

Cases were classified into three types according to cause of action, and the courts’ decisions and their grounds were analyzed from a legal perspective (Fig. 2).

In Type 1 cases, the plaintiff argued that PR was applied unnecessarily or excessively (*n* = 21). Courts ruled 12 cases of “PR with cable ties,” “PR accompanied by beatings,” “PR in situations where consent was not met,” and “PR due to staff shortage” as being inappropriate. However, in nine cases, the use of PR was considered appropriate because the care recipients had a high risk of falling or self-removal of medically necessary devices. Some courts also ruled that the use of PR was appropriate owing to staff shortages. Regarding duty of care, the courts’ decisions were divided according to compliance with PR use guidelines.

In Type 2, the plaintiff claimed that an accident occurred to the care recipients because PR was necessary but not applied (*n* = 11). The court ruled that four health workers were at fault for not using PR on care recipients exposed to risks such as falls, and for not performing risk-prevention interventions. For seven cases in which the care recipients’ condition was stable and a one-on-one sitter was present to prevent accidents, the courts ruled that the decision not to use PR was appropriate because PR should be used as a last resort. However, two of these seven cases were recognized as violations of duty of care because accidents occurred to the care recipients.

Type 3 cases are those in which the plaintiff claimed that accidents occurred to care recipients because health workers did not manage the applied PR properly (*n* = 6). In two cases, the courts found that health workers violated their duty of care by neglecting supervision until care recipients were released from restraint and exposed to accidents. In four cases, despite the occurrence of adverse outcomes like falls or the self-removal of medical devices, the courts did not acknowledge the violation of the health worker’s duty of care because they faithfully followed the PR use guidelines, such as observing the care recipient every two hours and changing the restraint as required, and the interventions performed were documented in the medical record.

### Classification according to the four principles of biomedical ethics

The cases were classified into four groups based on the ethical principles of the analytical framework.

#### Principle of autonomy

Seven care recipients on whom PR was used without informed consent were identified. Four of these cases were judged to be negligent by the courts because PR was used on stable patients, cable ties were used as restraints, and the care recipients were physically beaten. One court regarded the act of applying PR without consent as intentional assault, unless performed during an emergency. Another court emphasized the need to obtain consent from the care recipient or their family, which includes detailing the circumstances and time at which the PR would be used.

#### Principle of non-maleficence

There were 22 cases of adverse outcomes for care recipients due to health workers’ decisions regarding PR. Adverse outcomes were classified as falls, self-removal of medical devices, and death. Unfortunately, four of the five deaths, occurred in patients who were physically restrained in psychiatric wards. The courts did not rule nine cases as being negligent on the grounds that health workers followed the PR use guidelines, and that sudden self-extubations were difficult to predict.

#### Principle of beneficence

In 16 cases, the courts ruled that health workers’ judgments on PR were illegal or negligent (12 PR use cases and four PR disuse cases). In the PR use cases, health workers used PR for unethical purposes, such as punishing care recipients or overusing it for their own convenience. Cases of PR disuse had a high risk of accidents for the care recipient; however, due to the absence of appropriate preventive measures, including PR, accidents such as falls and tracheal tube removal eventually occurred.

#### Principle of justice

There were eight cases in which one of the reasons for using PR was staff shortage. In three cases, the courts ruled that the use of PR due to staff shortages was inappropriate. However, the courts in the remaining five cases ruled that the use of PR due to staff shortages was unavoidable, and therefore appropriate.

### Courts’ decisions regarding ethical dilemmas

Next, we examine how the courts’ decisions regarding ethical dilemmas by cases were made (Table 3). It was divided into 6 cases (A-F) according to the ethical dilemma situation and the court’s decision.

**Table 3:** Ethical dilemma situation and court’s decision by cases

Case	Case information			Ethical Dilemma Situation	Court’s Decision
	Place	Care recipient	Consequence		
A	Nursing home	Older adults with dementia (nasogastric tube, indwelling urinary catheter)	Nothing happened	1	Compliance
B	Nursing home	Older adults with dementia	Fall	1,2	Compliance
C	Nursing home	Older adults with dementia	Nothing happened	1,2	Compliance
D	Nursing home	Older adults with dementia	Nothing happened	3	Violation
E	Nursing home	Older adults with dementia (nasogastric tube, indwelling urinary catheter)	Nothing happened	3	Compliance
F	Hospital	Older adults with deterioration of consciousness (peripheral intravenous line, arterial line)	Nothing happened	3	Compliance

#### Situation 1: should PR be used without consent for safety?

In three cases, PR was applied to older adults with dementia in a nursing home (Cases A, B, and C). In these cases, consent was not obtained, but the courts determined that the PR was used appropriately for a “good purpose.” These good purposes were the prevention of the risk of self-removal of the Levin tube and foley catheter (Case A) and the prevention of falls from wheelchairs or beds (Cases B and C). The documentation that showed that health workers tried to use minimum PR also influenced court rulings. In particular, the court in Case C noted that PR used without consent can be justified in an urgent situation, and that consent should be obtained after using PR.

#### Situation 2: is it appropriate to use PR without consent due to staff shortages?

Cases B and C in Situation 1 also correspond to Situation 2. In Case B, an older adult tied to a wheelchair with an elastic bandage fell while trying to get out of the wheelchair, and in Case C, a health worker tied the arm of an older adult with a high risk of falling to a bed to care for another older adult. In both cases, the courts acknowledged that the use of PR is inevitable, even without consent, for the protection of older adults owing to the nature of nursing homes, in which a limited number of health workers care for highly dependent older adults. In particular, the court in Case B positively evaluated the nursing home as hiring health workers in compliance with the staffing standards set by the law.

#### Situation 3: is it appropriate to use PR with consent due to staff shortages?

Two cases occurred in a nursing home (Cases D and E), and one occurred in a hospital emergency room (Case F). In Case D, the court ruled that the health worker who applied PR to the older adult was negligent when she was forced to perform a workload of two staff members. In other words, the legitimacy of PR due to staff shortages was not recognized in this case. However, the courts in Cases E and F found that PR was unavoidable, and therefore appropriate, owing to staff shortages caused by the nature of the location.

## Discussion

Our study identified characteristics for the legitimate and safe use of PR by examining lawsuit cases through the lens of the four principles of biomedical ethics. Sharifi et al. [36], in an integrated literature review, underscored the significance of PR guidelines, patient monitoring, and obtaining informed consent. Similarly, Perez et al. [11] emphasized protocol compliance and the role of nurses in PR. These findings closely align with our research outcomes. Our study provides additional empirical and comprehensive data by analyzing real cases and adopting a legal and ethical approach.

This study confirmed the importance of determining the need for PR and when it should be used. However, the problem is that the criteria for determining these are unclear [37]. In general, the results of many studies acknowledge that PR is necessary in situations in which the risk of falling is high or cooperation in maintaining an indwelling medical device is difficult [36]. Decision-making tools can be used to make more objective judgments [38, 39]. According to one study, the use of the Restraint Decision Tree, which takes the patients’ muscle strength, delirium, and indwelling catheter levels into consideration, could reduce the indiscriminate use of PR [38]. Some studies have suggested that physicians should be involved in determining the need for PR [36, 40]. Unfortunately, doctors typically only prescribe the use of PR and are indifferent to PR decision-making [38, 41]. Various health workers must be aware of the ethical and legal issues regarding PR, and it is necessary to create a structure in which the decision for PR is discussed as a team, rather than being left to frontline staff.

Using unnecessary PR is a violation of the principle of beneficence and is clear “abuse.” According to a study on people with intellectual disabilities, abuse occurs not only because of the characteristics of the perpetrator and victim but also because of the organizational environment [42]. Social psychology emphasizes that ethical behavior is influenced more by situations than by human nature [43]. Our study supports the findings of these previous studies and theories. In particular, caring for people with low cognitive abilities makes ethical behaviors more difficult. To prevent the unethical use of PR, it may be helpful to improve the knowledge and attitudes of health workers by providing education on human rights and PR use guidelines [19, 44]. In addition, leadership is needed to create an organizational culture that does not permit unethical behavior by health workers [37, 45].

Another thing to pay attention to in terms of the principle of beneficence is the suitability of the restraining device. The court judges in this study used “easily removed in an emergency” as a criterion for determining device suitability according to Korean law, which is similar to previous studies conducted in other countries [36, 46]. However, the expression “not easily removable” in the commonly used definition of PR [1] prompts confusion about which devices are appropriate. Lethal adverse effects of PR, such as death, occur in relation to the restraint device, restraint position, and immobility [47, 48]. To reduce the fatal adverse effects caused by PR, it is necessary to develop a physiologically safe restraint device and position. However, most studies on PR seem to be more focused on ‘how to reduce the use of PR’ rather than ‘how to use PR well.’ It is already known that PR causes physical and psychological harm to patients [47–49], so we should minimize the use of PR. However, from the perspective of health workers, who must take responsibility for their patients’ safety, it is difficult to completely eliminate PR in the field [19, 50]. In other words, as long as there are even a small number of cases in which PR is needed, empirical studies on the safe use of PR should continue.

One interesting result is that the courts’ decisions on the use of PR due to staff shortages (Ethical dilemma situation 2 and 3) were different. Staff shortages have been identified as a factor that drives the use of PR, regardless of the care recipient’s condition [51, 52], and this is generally considered unethical situation, violating the principle of justice [16, 36]. However, studies conducted in Iran have shown that in situations of staff shortage, staff suffer ethical distress because there is no alternative for patient safety other than PR [52]. Frontline healthcare workers do not have the authority to address staff shortages. Enforcement measures, such as laws, may be necessary to ensure that the heads of institutions hire enough staff [53]. In South Korea, the staffing standards of hospitals and nursing homes are stipulated by law; however, the standards are too low and enforcement is weak [54]. Prior to legislation, the rationale for appropriate staffing standards should be established according to care recipient and institutional characteristics.

Health workers have a legal duty to pay attention and prevent harm to patients [55], aligning with the principle of non-maleficence. In the PR-related lawsuit analyzed in this study, adherence to guidelines emerged as the pivotal factor influencing the determination of whether the ‘duty of care’ was violated. Regular monitoring of care recipients who are either currently using or likely to use physical restraints is crucial for ensuring compliance with guidelines [36, 48]. Specific monitoring measures were identified in our study (e.g., checking restrained body parts every two hours and periodically releasing restraints). Healthcare workers might unintentionally miss monitoring responsibilities due to their demanding workloads. Using visual or auditory reminders to regularly reassess restrained patients could help address this challenge. Documentation is also important [51]. When a conflict arises, health workers are burdened with proving that their practices were appropriate [36, 48]. Even with diligent adherence to guidelines, health workers may face challenges in having their compliance recognized in a dispute without detailed documentation. In the United States, intensive care PR guidelines recommend thorough documentation in the medical record, covering the necessity for PR assessment, alternative interventions, and patient monitoring results [46].

Because the use of PR presupposes a violation of the principle of autonomy [19], consent can be a way to avoid this dilemma. The courts’ emphasis on detailed explanations and consent was consistent with the Helsinki Declaration of Informed Consent. Additionally, courts have ruled that the use of PR without consent (Ethical dilemma situation 1) is not illegal if it is for good purposes in emergency situations, which justifies paternalism. However, the justification for paternalism remains controversial and sometimes, care recipients may question practices based on paternalism [56–59]. Therefore, health workers must clarify that they have no choice but to use PR and obtain informed consent as soon as possible [36]. To avoid unnecessary controversy, we suggest that health workers who are likely to use PR should obtain consent for PR in advance.

**Fig. 3:**
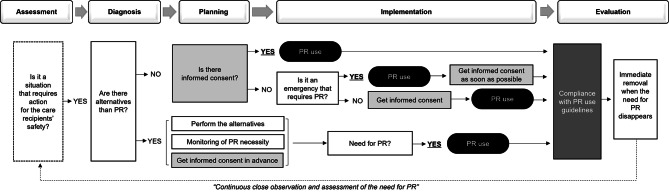
Flowchart of the legal and ethical decision for physical restraints according to the nursing process. *Note*: PR = physical restraint

Synthesizing the presented information using the nursing process yields an algorithm for PR use (Fig. 3). This guide helps health workers make informed decisions, ensuring the safety of care recipients and addressing ethical dilemmas related to PR.

This study has several limitations. The generalizability of the results is limited owing to the small sample size. Additionally, written legal judgments that were the main source of this study have their own limitations. Because sufficient information on the actual situations were not provided in the written judgments, it was difficult to extensively review and analyze situations that occurred in clinical practice. The legal judgment, written without medical input, lacks consideration for crucial professional factors in PR use, limiting in-depth practical and healthcare analysis. Future studies by healthcare professionals building upon our findings will be crucial for the safe use of PR. Nevertheless, this study provided empirical information on the use of PR using written judgments that most objectively described cases in the clinical field that were not easily accessible. In addition, it contributes to a practical understanding of the legitimate use of PR in clinical settings by extensively handling cases in various places in which PR is used and presenting an integrated ethical and legal perspective.

## Conclusion

PR remains controversial. Therefore, it is possible that health workers who use PR, as in the cases examined in this study, may be involved in difficult disputes. We analyzed lawsuit cases according to the four principles of bioethics and examined strategies for using PR from ethical and legal perspectives. Based on these results, health workers are expected to be able to make legal and ethically compliant decisions regarding PR use to ensure their own safety and that of their care recipients. Additionally, although efforts should be made to reduce the use of PR, which restricts a person’s autonomy, there are still situations in which PR is necessary for beneficence. Future studies exploring methods for using PR appropriately should be conducted.

### Electronic supplementary material

Below is the link to the electronic supplementary material.

Supplementary Material 1

## Data Availability

The datasets analysed during the current study are available in the “Written Judgment Management System” run by the Supreme Court of South Korea, https://www.scourt.go.kr/portal/information/finalruling/guide/index.html.

## Abbreviations

PR: Physical restraint
