# Supplementary material for: Is physical restraint unethical and illegal?: a qualitative analysis of Korean written judgments
Source: BMC Nurs. 2024 Feb 4;23:94. doi: 10.1186/s12912-024-01781-8 (PMC10838439; doi:10.1186/s12912-024-01781-8)
Supplement: Supplementary file 1 — Supplementary Material 1 [file 12912_2024_1781_MOESM1_ESM.docx]

**Supplemental material**

Three categories as elements for written judgments analysis

| **Category** | **Information extracted from the written judgments** | |
| --- | --- | --- |
| 1. Basic information about the lawsuit | a. Case number | e.g., 2015Na2015007 |
|  | b. Case type | - 1. Criminal case   2. Civil case |
|  | c. Trial stages | 1. First instance (district court) 2. Appeal (high court) 3. Final appeal (supreme Court) |
|  | d. Cause of action (i.e., plaintiff's claim) | |
| 2. Basic information about the case | a. Year the incident occurred | |
|  | b. Place where the incident occurred | |
|  | c. Whether informed consent was obtained at the time of the incident | |
|  | d. Characteristics of the care recipient (at the time of the incident) | 1. Age and sex 2. Reasons for entering the place where the incident occurred 3. Condition   - Impairment of autonomous judgment ability (e.g., dementia, mental illness, brain damage, decreased consciousness)  - Possession of artificial airway or various lines, and tubes (e.g., central venous catheter, L-tube, endotracheal tube) |
|  | e. Characteristics of frontline health worker | 1. Health worker on the frontline who had a duty to care for the care recipient at the time of the incident (i.e., physical restraints decision-maker) 2. Frontline health worker's decision regarding the use or non-use of physical restraints, and any accompanying interventions provided 3. Reason why the frontline health worker made the above decision 4. Whether there was any harsh treatment other than physical restraint (e.g. beating, confinement) |
|  | f. Direct impact of the frontline health worker’s decision and intervention on physical restraint use on care recipients (e.g., death, fall) | |
| 3. Court's decision | a. About the frontline health worker's decision regarding the use/or non-use of physical restraints and interventions (i.e., appropriateness of the frontline health worker's decision regarding physical restraints)  b. About the frontline health worker's violation of duty of care | |
